# A method for considering road curvature’s impact on aggressive turning

**DOI:** 10.1371/journal.pone.0338134

**Published:** 2025-12-05

**Authors:** Kai Liu, Haixia Feng, Zhixin Xu, Meng Guo, Xinsen Fan, Qiuxia Li, Zhongke Feng

**Affiliations:** 1 School of Transportation and Logistics Engineering, Shandong Jiaotong University, Jinan, Shandong, China; 2 Beijing Key Laboratory of Precision Forestry, Beijing Forestry University, Beijing, Beijing, China; Chang'an University, CHINA

## Abstract

Addressing the current issue of not considering the influence of road geometry in the recognition of aggressive turning, a dangerous driving behavior, this paper proposes a method to eliminate the impact of road curvature on the recognition of aggressive turning based on On-Board Diagnostics (OBD) trajectory data and high-precision electronic map data. The method is validated using Jinan City as the study area. The results show that the proposed method effectively removes the influence of road curvature on the recognition of aggressive turning (left or right turns), reduces misjudgments of dangerous driving behaviors of drivers, and enhances the accuracy of identifying drivers’ aggressive turning hazardous behaviors.

## Introduction

Road traffic safety involves multiple aspects including people, vehicles, roads, and the environment. Among them, drivers serve as a crucial factor in the road traffic system, and their driving behavior has a significant impact on road traffic safety [1,2]. Dangerous driving behaviors during the driving process can create hidden dangers for road traffic safety and even lead to traffic accidents [3–5]. According to data from the World Health Organization, 90% of traffic accidents are caused by human factors, and abnormal or dangerous driving behaviors of drivers are triggers for some major traffic accidents [6–8]. Dangerous driving behaviors encompass sudden acceleration, rapid acceleration and deceleration, continuous lane changing, sharp turns, fatigued driving, emotional driving, and more [9,10]. The precise identification of dangerous driving behaviors is of utmost importance for road traffic safety [11,12].

Data from various modalities, such as eye trackers, video images, and vehicle trajectories, are gradually being applied to the identification of dangerous driving behaviors. For instance, Sun [13] Liu [14] and Li [15] and others have constructed recognition models for unsafe driving behaviors based on video data and facial features of drivers, respectively. Chandrasiri [16], Zhao [17], Li [9] and others have established driving behavior recognition models based on trajectory data. Multimodal data have been used to recognized dangerous driving behaviors [18,19]. Techniques such as Support Vector Machines [20,21], Feature engineering [22], Neural networks [23,24] and Attention mechanism [25] are also increasingly being applied to the recognition of driving behaviors.

Sharp turns belong to a type of dangerous driving, but some sharp turns in actual driving are caused by road geometry, such as when vehicles must make sharp turns at road curves with large curvature (where the road alignment forms a circular curve with a large radius). This has nothing to do with the driver’s driving behavior or habits. The influence of road geometry and alignment factors on driving behavior is gradually being recognized. For example, Yang et al. developed a car-following model that considers road geometry [26]; Bakibillah et al. proposed a driving strategy to reduce fuel consumption and emissions on curved roads [27]; Goyani et al. analyzed the impact of curve geometry factors on drivers—speed decisions when navigating horizontal curves [28]; Jia et al. introduced a novel nonlinear drift control method for vehicles making sharp turns [29].

With the continuous improvement in the accuracy of electronic maps and the rapid development of information technology, navigation and positioning have become essential choices for most vehicle drivers. The combination of trajectory data and high-precision electronic maps to identify driving behavior is becoming a trend. For instance, Vergara et al. analyzed the impact of interstate horizontal curves and slopes on speeding behavior using comprehensive detection data [30], while Li et al. quantified the risk of lane-changing behavior on highways using vehicle trajectory data from different driving environments [9]; Zhao et al. conducted dangerous driving trajectory identification using trajectory data, including abnormal trajectory detection, lane-changing recognition, and speed anomaly identification [31]; Li et al. developed an LSTM-based crash risk prediction model from trajectory data, identifying high-risk driving behaviors on highway segments and intersections [32]; Dong et al. proposed a trajectory data-driven approach for freeway crash risk assessment [33];Yuan et al. created a CNN-LSTM model for traffic conflict prediction using microscopic vehicle trajectories, identifying risk factors leading to dangerous merging behaviors [34]. On-Board Diagnostics (OBD) data not only provides vehicle positioning information but also offers information on the working status of various systems and components of the vehicle, making it an important data source for driving behavior recognition. Kumar and Priyadharshini have separately conducted driving behavior analysis and evaluation using vehicle OBD data [35,36]. However, research on how to eliminate the influence of road alignment factors in the identification of sharp turns as a dangerous driving behavior has not been retrieved. This paper intends to utilize On-Board Diagnostics (OBD) trajectory data and high-precision electronic map data to eliminate the influence of road alignment itself on the identification of sharp turns as a dangerous driving behavior, thereby enhancing the accuracy of distinguishing dangerous driving behaviors among drivers.

## Data and methods

### Study area

**Fig 1:**
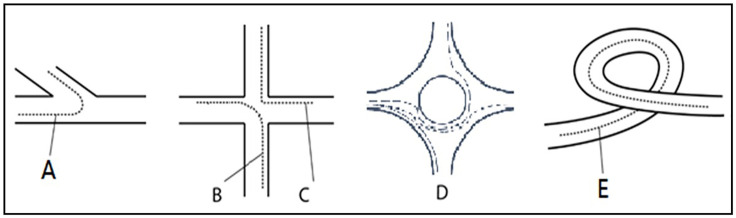
Types of sharp turn. Types of sharp turn. A, B, C, D, and E represent sharp-turn conditions requiring consideration of road alignment effects.

This paper takes Jinan City in Shandong Province as the experimental area. Jinan, the capital of Shandong Province, belongs to the warm temperate continental monsoon climate zone, as shown in Fig 1. As of August 2024, the total number of motor vehicles in the city has exceeded 3 million, reaching 4.04 million, including 3.472 million cars, leading to relatively severe traffic congestion. In 2016 and 2018, Jinan ranked first nationwide in terms of annual commute peak congestion levels. Although the situation has eased in recent years, traffic congestion remains a serious issue.

### Data

The primary data used in this paper mainly fall into three categories: OBD data, high-precision electronic map data, and remote sensing imagery. We have built a vehicle condition monitoring platform equipped with the OBD system, currently with over 200 vehicles online, primarily cars and some maintenance vehicles on highways. The OBD data used in this paper originates from our own platform, with each piece of OBD information including vehicle ID, timestamp, longitude and latitude per second during vehicle operation, vehicle speed, acceleration, braking status, steering angle, and other data. This paper utilizes OBD data from cars collected between January and June 2024, primarily focusing on longitude and latitude per second, vehicle speed, and steering angle data during vehicle operation. The high-precision map utilizes electronic map data from Gaode Navigation (Amap, https://www.amap.com/), which includes detailed and accurate road network information for Jinan City. AMap APP is the most widely used navigation software in China. Users can download offline map data within the AMap APP or directly import trajectory files into the APP. For a clearer representation of the road network and its surroundings, the display utilizes an image map based on remote sensing data from Landsat (https://landsat.visibleearth.nasa.gov/). For a clearer representation of the road network and its surroundings, the display utilizes an image map based on remote sensing data from Landsat (https://landsat.visibleearth.nasa.gov/). Remote sensing imagery of Jinan City, obtained from the Landsat website (https://landsat.visibleearth.nasa.gov/).

The positioning data (longitude and latitude) were cleaned and reconstructed to remove abnormal and redundant data, and then matched with the map. The trajectory data and the matching were corrected using the trajectory and road network.

The change in the vehicle’s heading angle per second $\varpi_{t}$ was calculated, which is the absolute value of the change in azimuth angle between the next moment $\varpi_{2}$ and the previous moment, denoted as $\varpi_{t}=\left|\varpi_{2}-\varpi_{1}\right|$.

### Methods

#### Situation determination for sharp turn with road alignment influence eliminated.

In general, sharp turn is identified when the instantaneous speeds$\;v_{1}\;$and $v_{2}$ at moments$\;t_{1}$ and $t_{2}$ are both greater than 30 km/h, and the absolute value of the change in azimuth angle between these two moments $\varpi_{t}$ is ≥ 15°. Sharp turning typically occurs in four scenarios: vehicle U-turn (generally a 180° sharp turn), vehicle lane change, vehicle left or right turn, and road sharp turn (a curve with a large curvature in the road plane). Vehicle U-turns and lane changes do not require consideration of road alignment, but vehicle left or right turns and road curves (see Fig 1) do require consideration of road alignment.

When a vehicle is determined to be in a sharp turn, the road centerline of the road where the sharp-turning vehicle is located is called. The possible shapes of the road centerline during left or right turns and road sharp turns are shown in Fig 3, along with the possible trajectory lines during sharp turns.

**Fig 2:**
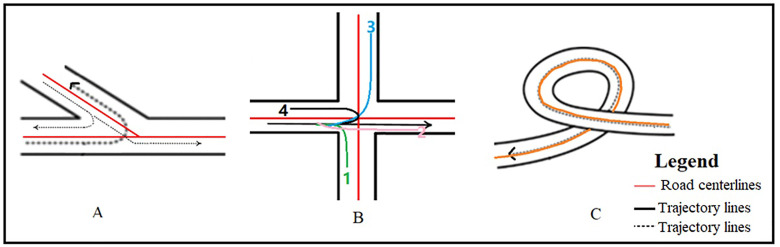
Road centerlines and sharp turn trajectory. A, B, and C demonstrate the spatial relationships between vehicle trajectories and road centerlines during sharp-turn maneuvers.

If the intersection road centerline angle is 90°; and the direction change of the trajectory line is 0 < 30° or 160° < 180° < 200°, it is determined as a vehicle lane change (Fig 2B) or vehicle U-turn (Fig 2B), and road alignment does not need to be considered. If the direction change of the trajectory line is 80° < 90° < 100°, it is determined as a vehicle left or right turn (Cases 1 and 3 in Fig 2B), and road alignment needs to be considered. If the intersection road centerline angle ≠ 90° (Fig 1A, C, D, E), road alignment needs to be considered.

#### Method for eliminating road alignment influence in sharp turning.

From Figs 1 and 2 and the sharp turning determination method, eliminating the influence of road alignment on sharp turning mainly involves eliminating the influence of road curvature, then, after eliminating the curvature angle α, the turning angle ϖ is:


$$\varpi =\varpi_{t}-\alpha$$ (1)

Where ϖ is the turning angle after eliminating the influence of curvature angle, $\varpi_{t}$ is the vehicle’s turning angle at two moments, i.e., $\varpi_{t}=\left|\varpi_{2}-\varpi_{1}\right|$ and α is the curvature angle caused by road curvature, is as follows:


$$\alpha =\frac{L}{R}$$ (2)

Where, α represents the angle of turn caused by the road alignment, R is the curvature radius of the road centerline, and L represents the travel distance of the vehicle per second.

The turning radius R of urban road intersections (calculated based on the road red line) is controlled according to the following standards: 20m–30m for main roads, 15m–20m for secondary roads, 10m–20m for non-primary and secondary roads, no less than 6m for residential area road red lines, no less than 9m for industrial areas, and a minimum turning radius of 12m for roads with fire protection functions.

Therefore, the corrected turning angle ϖ (with curvature compensation) is computed as:


$$\varpi =\varpi_{t}-\alpha =\left|\varpi_{2}-\varpi_{1}\right|-\frac{L}{R}$$ (3)

Where, the unit of α needs to be consistent with ϖ.

Therefore, the sharp-turn detection criterion incorporating road curvature effects is:


$$\{\omega \geq 15^{\circ }v(t_{1}),v(t_{2})\geq 30km/h$$ (4)

### Results

**Fig 3:**
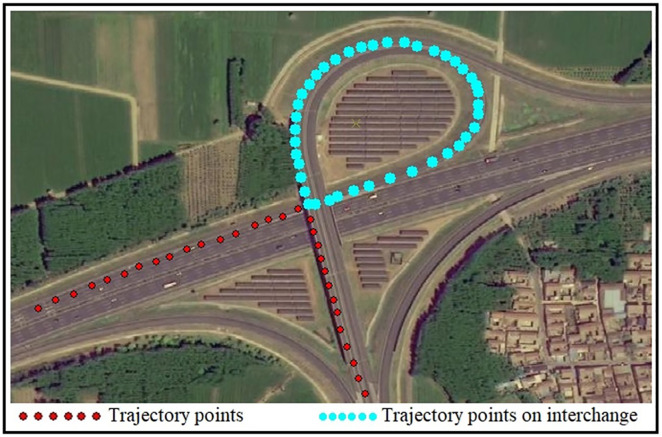
Vehicle Trajectory (Zhangqiu Interchange). The base map is a remote sensing image of the area near Zhangqiu Interchange in Jinan. The red dots represent vehicle trajectory points, green areas indicate vegetation, and the interchange connects to the Qinyin Expressway.

This article analyzes the OBD (On-Board Diagnostics) data of Car traveling on Zhangqiu Interchange at 10:10 AM on January 1, 2024, using it as an example. The trajectory data is illustrated in Fig 3.

When passing through the interchange (light brown, its second-by-second speed, curvature angle α, and post-curvature angle α, as well as the turning angle, are listed in Table 1.

**Table 1:** Second-by-second Turning Angles.

Trajectory point	Longitude	Latitude	Vehicle speed v (km/h)	$\varpi_{t}$ (°)	α(°)	ϖ (°)
**1**	117.4862	36.86051	54	1	0.57	0.43
**2**	117.4861	36.86064	54	1	0.60	0.40
**3**	117.4861	36.86077	51	0	0.64	−0.64
**4**	117.4861	36.8609	51	1	0.67	0.33
**5**	117.486	36.86102	50	1	0.72	0.28
**6**	117.486	36.86114	48	2	0.76	1.24
**7**	117.486	36.86138	47	5	0.82	4.18
**8**	117.4861	36.86151	48	4	0.88	3.12
**9**	117.4861	36.86151	47	1	0.95	0.05
**10**	117.4861	36.86162	47	4	1.04	2.96
**11**	117.4862	36.86174	47	4	1.15	2.85
**12**	117.4862	36.86186	48	3	1.27	1.73
**13**	117.4863	36.86195	47	8	1.43	6.57
**14**	117.4864	36.86204	47	6	1.55	4.45
**15**	117.4866	36.86214	47	6	1.79	4.21
**16**	117.4867	36.86222	46	5	1.91	3.09
**17**	117.4868	36.86231	46	7	2.29	4.71
**18**	117.487	36.86239	44	9	2.60	6.40
**19**	117.4872	36.86233	43	8	2.86	5.14
**20**	**117.4874**	**36.8623**	**42**	**25**	**3.18**	**21.82**
**21**	117.4875	36.86225	41	10	3.58	6.42
**22**	117.4877	36.86214	39	14	4.09	9.91
**23**	117.4878	36.86211	39	**15**	4.09	10.91
**24**	117.4879	36.86204	39	**16**	4.76	11.24
**25**	117.4879	36.86204	40	**19**	7.13	11.87
**26**	117.4879	36.86184	40	**24**	9.46	14.54
**27**	**117.4879**	**36.86174**	**41**	**27**	**11.31**	**15.69**
**28**	**117.4879**	**36.86167**	41	**20**	7.13	12.87
**29**	117.4879	36.86158	42	**15**	2.86	12.14
**30**	117.4879	36.8615	42	8	2.39	5.61
**31**	117.4878	36.86144	43	9	1.91	7.09
**32**	117.4875	36.86128	45	8	1.15	6.85
**33**	117.4874	36.8612	45	5	1.04	3.96
**34**	117.4873	36.86112	46	2	0.95	1.05
**35**	117.4873	36.86112	47	0	0.88	−0.88
**36**	117.4871	36.86104	47	2	0.82	1.18
**37**	117.487	36.86096	48	2	0.76	1.24
**38**	117.4869	36.86089	48	3	0.72	2.28
**39**	117.4867	36.8608	48	0	0.67	−0.67
**40**	117.4866	36.86073	49	1	0.64	0.36
**41**	117.4865	36.86066	49	1	0.59	0.41
**42**	117.4863	36.86061	50	2	0.57	1.43

During the passage over the interchange (highlighted in bright blue), the second-by-second speed v, the vehicle turning angle $\varpi_{t}$, the steering angle α caused by road curvature, and the turning angle ϖ after eliminating the influence of road curvature α are presented in Table 1.

**Fig 4:**
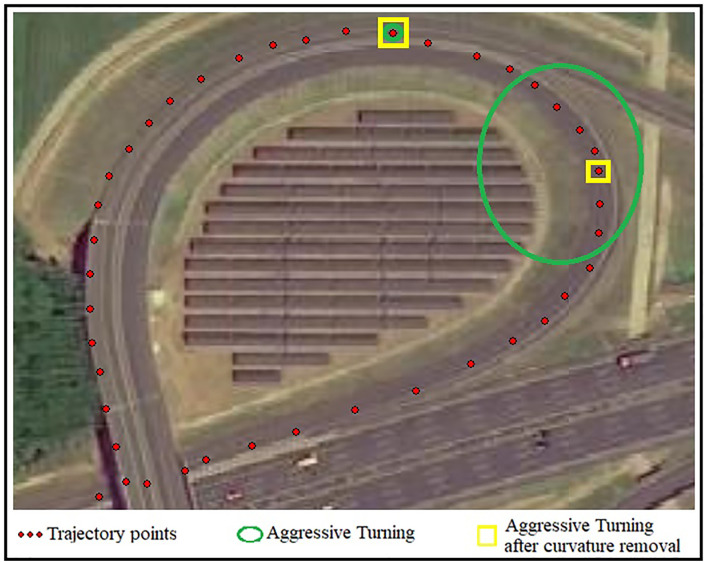
Sharp Turning Points. Red dots represent vehicle trajectory points. Green circles indicate points meeting the sharp-turn criteria. Yellow rectangles mark points identified as sharp turns after road curvature effects were removed.

**Fig 5:**
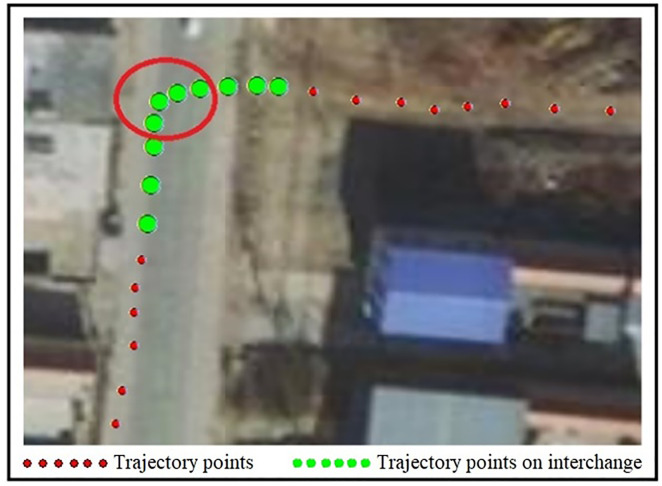
Left Turn. Red points represent trajectory data, while green points indicate trajectory points affected by road alignment.

According to Table 1, when passing through Zhangqiu Interchange, the instantaneous speeds at all 42 trajectory points are above 30 km/h. A total of 8 points have turning angles exceeding 15°, as indicated by the green circles in Fig 5, which meets the criteria for sharp turning driving behavior. However, considering the influence of the road alignment of Zhangqiu Interchange on vehicles and removing the effect of road curvature on turning angles, only points 20 and 27 meet the criteria for sharp turning, as shown in the yellow boxes in Fig 4.

As seen in Fig 4, point 20 represents a sharp turn caused by the need for emergency lane changes at a fork in the road; point 27 is due to a turn from the outer lane to the inner lane when the road curvature is large. After removing the influence of road curvature, the other 6 turning points are no longer considered as sharp turning driving behavior.

Taking the OBD data of Car 2 during a left turn at 11:05 AM on January 21, 2024, as an example for analysis, its trajectory data is shown in Fig 5.

As seen in Fig 5, due to the low traffic volume on the rural road, the vehicle speed is relatively fast. During the left turn, its second-by-second speed v, the vehicle turning angle , the steering angle α caused by road curvature, and the turning angle ϖ after eliminating the influence of road curvature $\varpi_{t}$α are presented in Table 2.

**Table 2:** Second-by-second Turning Angles.

Trajectory point	Longitude	Latitude	Vehicle speed (km/h)	ωt(°)	α(°)	ω(°)
**1**	117.2352	36.77968	37	0	0	0
**2**	117.2351	36.77969	36	1	0	1
**3**	117.2351	36.77968	35	2	0.72	1.28
**4**	117.2351	36.77968	35	28	26.57	1.43
**5**	117.2351	36.77968	25	32	26.57	5.43
**6**	117.235	36.77966	31	35	26.57	8.43
**7**	117.235	36.77962	37	29	26.57	2.43
**8**	117.2349	36.77959	38	2	0.72	1.28
**9**	117.2349	36.77954	39	2	0.00	2.00
**10**	117.2349	36.77949	40	0	0.00	0.00

According to Table 2, when passing through the intersection, except for point 5, the instantaneous speeds at the other 9 trajectory points are above 30 km/h. A total of 4 points have turning angles exceeding 15°, as indicated by the red circles in Fig 5, which meets the criteria for sharp turning driving behavior. However, considering the influence of road alignment (with a road centerline angle of 90°) on vehicles and removing the effect of road curvature on turning angles, there are no points that meet the criteria for sharp turning driving.

## Discussion

Accurate identification and early warning of dangerous driving behaviors are crucial for reducing traffic accidents and ensuring traffic safety. Sharp turns, as a dangerous driving behavior, are not always the driver’s intention but can be caused by road geometry. This method proposed in this paper takes into account the influence of road alignment itself on sharp turn angles, making it applicable to scenarios such as sharp curves, left/right turns at intersections, and U-turns, thereby reducing misjudgments of sharp turns as dangerous driving.

In scenarios involving sharp curves or turns at intersections (e.g., left/right turns or U-turns), the turning maneuver is necessary and not the driver’s voluntary choice. If the influence of road alignment is ignored, misjudgments of sharp turns as dangerous driving may occur. For example, when a vehicle passes the Zhangqiu Overpass (see Fig 4), without accounting for road curvature, eight trajectory points would be classified as sharp turns based on the current criteria (steering angle exceeding 15° between consecutive seconds and speed exceeding 30 km/h). However, using the proposed method—which removes the influence of the overpass’s inherent curvature—only two trajectory points are identified as sharp turns. Similarly, for Vehicle 2 during a left turn (Fig 5), the existing method labels four trajectory points as sharp turns, whereas the proposed method, after eliminating road curvature effects, identifies no sharp turn behavior. The proposed method effectively removes the influence of road alignment on sharp turn detection, thereby improving the accuracy of identifying dangerous driving behaviors involving sharp turns.

The unit of curvature angle α caused by road alignment, i.e., curvature, is π, while the unit of turning angle is degrees. Therefore, when considering removing the influence of road alignment on the turning angle, the unit of α needs to be consistent with that of the turning angle, i.e., converting π to degrees.

The OBD data used in this paper are collected on a second-by-second basis. Therefore, when calculating the curvature angle resulting from road alignment, i.e., curvature, Y represents the vehicle’s travel distance for each second. The travel distance in this paper is the product of instantaneous speed and time, which does not fully align with the actual travel distance (the arc length of the curve) when the curvature is significant.

In the proposed method for eliminating the influence of road curvature on sharp-turn driving behavior identification, the road curvature is derived from the centerline curvature of roads. Current electronic map precision generally meets the computational requirements. For intersections (e.g., cross intersections and T-junctions), road curvature is estimated based on the centerlines in electronic maps. The interchange is more complex due to variable curvature across different segments: the ramps may employ circular curves, and the mainlines may use transition curves (with linearly varying curvature), or composite curves (with continuous but non-linear curvature variation). The *Chinese Industry Standard: Highway Route Design Specification* (JTG D20-2017) specifies geometric indices for mainlines within interchanges (see Table 3).

**Table 3:** Geometric Design Criteria for Mainlines within Interchange Areas.

Design Speed (km/h)			120	100	80	60
**Minimum radius of circular curve (m)**		general value	2000	1500	1100	500
		absolute minimum	1500	1000	700	350
**Minimum vertical curve radius (m)**	convex	general value	45000	25000	12000	6000
		absolute minimum	23000	15000	6000	3000
	concave	general value	16000	12000	8000	4000
		absolute minimum	12000	8000	4000	2000

In current interchange design, clothoid curves (also called Euler spirals) are predominantly used for transition curves. Their mathematical expression demonstrates an inverse relationship between radius (R) and curve length (L). This alignment design achieves continuous curvature transition, effectively balances centrifugal force variation, and enhances both ride smoothness and safety. Access to original interchange design parameters (e.g., A, R, L values) during implementation would significantly enhance the method’s accuracy.

The proposed method is theoretically applicable to other vehicle types. However, the validation data in this study were exclusively obtained from our team’s self-developed OBD-based vehicle condition monitoring platform, which currently only collects data from passenger cars. Consequently, verification for other vehicle types was not performed. Furthermore, as no appropriate methods were identified for comparison, the analysis only considered the difference between using and not using the proposed method. The limitations of the proposed method in scalability and benchmarking also define directions for future work.

Modern real-time navigation systems can generally provide all required data for this algorithm, including position, speed, steering angle, and road curvature (derived from digital map road data). Therefore, the algorithm can be readily integrated with in-vehicle navigation systems, enabling synchronous data acquisition from in-vehicle navigation systems for driving behavior monitoring. Due to the authors’ lack of system development experience, practical experiments on the application of this method in traffic safety were not conducted. We welcome navigation platforms, safe driving platforms, and other interested parties to verify and provide feedback on the practicality of the proposed method.

The method proposed in this paper, to a certain extent, eliminates the influence of road alignment on the discrimination of drivers’ sharp turning behavior, enhancing the accuracy of assessing drivers’ driving behavior. However, the risk associated with vehicles making sharp turns remains an objective reality.

## Conclusion

This paper addresses the issue of not considering the influence of road alignment in current sharp turning judgments. We propose a novel method integrating OBD trajectory data with map data to eliminate road alignment effects the determination of sharp turning. After verification, the results show that Our approach effectively removes the influence of road curvature on the recognition of aggressive turning (left or right turns), reduces behavioral misjudgment of drivers’ behaviors. This study can achieve precise monitoring of drivers’ dangerous driving behaviors. This work advances traffic safety while promoting integrated applications of trajectory and map data, offering new solutions for traffic management.

## References

[1] Gao Y, Gong J. Characteristics and mechanism of road traffic accidents. J Saf Environ. 2023;23(11):4013–23.

[2] Liu W, Li H, Zhang H. Dangerous Driving Behavior Recognition Based on Hand Trajectory. Sustainability. 2022;14(19):12355. doi: 10.3390/su141912355

[3] Wang J. Development of a society on wheels: Understanding the rise of automobile-dependency in China. Singapore: Springer; 2019. p. 211–32.

[4] Tong Z, Dan Q, Wen W. Research on accident risk identification and influencing factors of bus drivers based on machine learning. China Saf Sci J. 2023;33(2):23.

[5] Feng Y, Zhang L. Analysis on mediating effect of dangerous driving behavior between distraction and accident. J Saf Sci Technol. 2024;20(8):210–6.

[6] Trirat P, Lee J-G. DF-TAR: A Deep Fusion Network for Citywide Traffic Accident Risk Prediction with Dangerous Driving Behavior. In: Proceedings of the Web Conference 2021. 2021. p. 1146–56.

[7] Guo M, Zhao X, Yao Y. Study on accident risk based on driving behavior and traffic operating status. J South China Univ Technol (Natl Sci Edit). 2022;50(9):29–38.

[8] Hu L, Bao X, Wu H, Wu W. A Study on Correlation of Traffic Accident Tendency with Driver Characters Using In-Depth Traffic Accident Data. J Adv Transp. 2020;2020:1–7. doi: 10.1155/2020/9084245

[9] Li J, Ling M, Zang X, Luo Q, Yang J, Chen S, et al. Quantifying risks of lane-changing behavior in highways with vehicle trajectory data under different driving environments. Int J Mod Phys C. 2024;35(11). doi: 10.1142/s0129183124501419

[10] Ahmed J, Ward N, Otto J, McMahill A, Miller EE. Identifying Measures of Emotional Intelligence and Dangerous Driving. Transp Res Rec. 2023;2678(3):365–75. doi: 10.1177/03611981231179698

[11] Lu J, Wang K, Jiang YM. Real-time identification method of abnormal road driving behavior based on vehicle driving trajectory. J Traffic Transp Eng. 2020;20(6):227–35.

[12] Qu Z, Cui L, Yang X. HAR-Net: An Hourglass Attention ResNet Network for Dangerous Driving Behavior Detection. Electronics. 2024;13(6):1019. doi: 10.3390/electronics13061019

[13] Sun W, Zhang X, Peeta S, He X, Li Y. A Real-Time Fatigue Driving Recognition Method Incorporating Contextual Features and Two Fusion Levels. IEEE Trans Intell Transport Syst. 2017;18(12):3408–20. doi: 10.1109/tits.2017.2690914

[14] Liu T, Yang Y, Huang G-B, Yeo YK, Lin Z. Driver Distraction Detection Using Semi-Supervised Machine Learning. IEEE Trans Intell Transport Syst. 2016;17(4):1108–20. doi: 10.1109/tits.2015.2496157

[15] Li L, Zhang Q. Research on Visual Cognition About Sharp Turn Sign Based on Driver’s Eye Movement Characteristic. Int J Patt Recogn Artif Intell. 2017;31(07):1759012. doi: 10.1142/s0218001417590121

[16] Chandrasiri NP, Nawa K, Ishii A. Driving skill classification in curve driving scenes using machine learning. J Mod Transport. 2016;24(3):196–206. doi: 10.1007/s40534-016-0098-2

[17] Zhao J, Chen Q, Jiao Y. Recognition of abnormal driving behavior of key commercial vehicles. J Transp Syst Eng Inform Technol. 2022;22(1):282–91.

[18] Wu J, Zhang Z, Wang Y. Method for Identifying Dangerous Driving Behaviors in Heavy-Duty Trucks Based on Multi-Modal Data. J Transp Syst Eng Inform Technol. 2024;24(2):63–76.

[19] Hao Z, Li Z, Dang X, Ma Z, Liu G. MM-LMF: A Low-Rank Multimodal Fusion Dangerous Driving Behavior Recognition Method Based on FMCW Signals. Electronics. 2022;11(22):3800. doi: 10.3390/electronics11223800

[20] Liang Y, Reyes ML, Lee JD. Real-Time Detection of Driver Cognitive Distraction Using Support Vector Machines. IEEE Trans Intell Transport Syst. 2007;8(2):340–50. doi: 10.1109/tits.2007.895298

[21] Zhang L, Tan B, Liu T, et al. Research on recognition of dangerous driving behavior based on support vector machine[C]. Twelfth International Conference on Graphics and Image Processing (ICGIP 2020). SPIE; 2021:11720:471–6.

[22] Liu Y, Wang J, Zhao P, Qin D, Chen Z. Research on Classification and Recognition of Driving Styles Based on Feature Engineering. IEEE Access. 2019;7:89245–55. doi: 10.1109/access.2019.2926593

[23] Xiang H, Zhu J, Liang G, Shen Y. Prediction of Dangerous Driving Behavior Based on Vehicle Motion State and Passenger Feeling Using Cloud Model and Elman Neural Network. Front Neurorobot. 2021;15:641007. doi: 10.3389/fnbot.2021.641007 33994985 PMC8116708

[24] Bejani MM, Ghatee M. Convolutional Neural Network With Adaptive Regularization to Classify Driving Styles on Smartphones. IEEE Trans Intell Transport Syst. 2020;21(2):543–52. doi: 10.1109/tits.2019.2896672

[25] Liu J, Yang N, Lee Y, Huang W, Du Y, Li T, et al. FedDAF: Federated deep attention fusion for dangerous driving behavior detection. Inform Fus. 2024;112:102584. doi: 10.1016/j.inffus.2024.102584

[26] Yang X, Liu Z, Cheng Q, Liu P. Geometry-aware car-following model construction: Theoretical modeling and empirical analysis on horizontal curves. Transp Res Part C Emerg Technol. 2024;166:104772. doi: 10.1016/j.trc.2024.104772

[27] Bakibillah ASM, Kamal MAS, Tan CP, Hayakawa T, Imura J-I. Optimal eco-driving scheme for reducing energy consumption and carbon emissions on curved roads. Heliyon. 2023;10(1):e23586. doi: 10.1016/j.heliyon.2023.e23586 38173479 PMC10761797

[28] Goyani J, Mali R, Ramesh A, Saimadhu K, Elango S, Arkatkar S. Influence of Curve Geometry Factors on Driver’s Speed Decision Making When Passing through a Horizontal Curve. J Transp Eng, Part A Syst. 2024;150(1). doi: 10.1061/jtepbs.teeng-7919

[29] Jia F, Jing H, Liu Z. A novel nonlinear drift control for sharp turn of autonomous vehicles. Vehicle Syst Dyn. 2023;62(2):490–510. doi: 10.1080/00423114.2023.2180755

[30] Vergara E, Aviles-Ordonez J, Xie Y, Shirazi M. Understanding speeding behavior on interstate horizontal curves and ramps using networkwide probe data. J Safety Res. 2024;90:371–80. doi: 10.1016/j.jsr.2024.05.003 39251293

[31] Zhao L, Fu Z, Yang J, Zhao Z, Wang P. Multi-Adjacent Camera-Based Dangerous Driving Trajectory Recognition for Ultra-Long Highways. Appl Sci. 2024;14(11):4593. doi: 10.3390/app14114593

[32] Li H, Yu L. Prediction of traffic accident risk based on vehicle trajectory data. Traffic Inj Prev. 2025;26(2):164–71. doi: 10.1080/15389588.2024.2402936 39570198

[33] Dong C, Xu B, Li P. Trajectory data-driven risk assessment of freeway accidents. J Beijing Jiaotong Univ. 2024;48(6):12–21.

[34] Yuan R, Abdel-Aty M, Zhao Y, Xiang Q. Studying merge behaviour in weaving segments: insights from traffic conflict prediction and risk factors analysis. Transportmetrica A Transp Sci. 2025;1–26. doi: 10.1080/23249935.2025.2479085

[35] Kumar R, Jain A. Driving behavior analysis and classification by vehicle OBD data using machine learning. J Supercomput. 2023;1–20. doi: 10.1007/s11227-023-05364-3 37359337 PMC10198028

[36] Priyadharshini G, Ferni Ukrit M. An empirical evaluation of importance-based feature selection methods for the driver identification task using OBD data. Int J Syst Assur Eng Manag. 2022;1–12. doi: 10.1007/s13198-022-01695-1

